# Observed Instagram use and satisfaction with life: Associations with received communications and exploration of others’ content after posting a selfie

**DOI:** 10.1371/journal.pone.0297392

**Published:** 2024-03-27

**Authors:** Kyla M. Cary, Megan K. Maas, Kirsten M. Greer, Dar Meshi

**Affiliations:** 1 Department of Applied Health Science, School of Public Health-Bloomington, Indiana University, Bloomington, Indiana, United States of America; 2 Department of Human Development and Family Studies, Michigan State University, East Lansing, Michigan, United States of America; 3 Department of Advertising and Public Relations, Michigan State University, East Lansing, Michigan, United States of America; King Abdulaziz University, SAUDI ARABIA

## Abstract

Over 70% of Americans use social media platforms, like Instagram. With this high prevalence, researchers have investigated the relationship between social media use and psychological well-being. Extant research has yielded mixed results, however, as most measures of social media use are self-reported and focus on amount of use. Even when studies account for type of social media use, such as active or passive use, there remains much to be captured. To address this, we asked participants to report their satisfaction with life and then recorded their actual Instagram activity for 10 minutes after posting a “selfie” portrait to their account. We coded the observed Instagram activity into the following four clusters of experiences: communications received, communications sent, monitoring self-related content, and exploring other-related content. We found that greater life satisfaction was associated with higher frequency of receiving communications and viewing other-related content. Life satisfaction was not associated with frequency of sending communications and self-monitoring. Surprisingly, none of the clusters of Instagram experiences were negatively associated with life satisfaction. Our findings highlight the importance of objective data and moving beyond the active/passive dichotomy of social media use to consider additional experiences.

## Introduction

Approximately 72% of all Americans use at least one form of social media, and the prevalence of social media use in modern society is rising [1]. In the United States, Instagram is the second most used social media platform (after Facebook), with 63% of U.S. adults using Instagram daily and 42% using the application (app) multiple times throughout the day [2]. Emerging adults ages 18–24 years account for the largest group of Instagram users, with 76% of youth in this age group using the app regularly [3]. Instagram facilitates observations of and interactions with others, and the platform has been described as highly-visual due to its focus on sharing user-generated visual content [4]. Typical Instagram use consists primarily of photo and video sharing, with users regularly uploading images and videos of their daily lives for others, or “followers,” to view. The focus of image sharing on Instagram makes the platform somewhat unique from other social networking sites such as Facebook or Twitter, where sharing personal photos and videos is possible but not the central activity. With these platform characteristics and the high prevalence of use mentioned above, Instagram presents a distinct online context in which emerging adults interact with others over visual imagery. However, concerns about social media use, including Instagram use, have been raised within the media and research literature, leading to a growing body of research exploring social media use and well-being.

The following literature review and the current study draw upon theory highlighting the importance of social interaction to humans’ health, adjustment, and well-being. Baumeister and Leary’s (1995) need to belong theory posits that social connection is a powerful driver of human behavior stemming from evolution [5]. Our ancestors with genes that predisposed them to find social interactions rewarding reaped the benefits of group living (i.e., increased safety, access to sustenance, increased likelihood of reproductive success). As such, these pro-social genes were naturally selected over generations, leading to current humans having a strong need to socially connect with others and belong to a group. Relevant to the present study, need to belong theory argues that individuals who are unable to maintain social connections experience a variety of ill effects, including decreased subjective well-being [5]. In line with this, preference for solitude, or lack of social interaction, has been found to be negatively associated with life satisfaction across multiple age groups [6]. On the other hand, maintenance of social ties has a strong positive effect on subjective well-being [7]. In the current media saturated social landscape, social media platforms such as Instagram provide an environment to seek and fulfill the evolutionary need for social connectedness and belonging [8]. Indeed, literature supports that those who are lonely are likely to seek social interaction within online spaces such as social media [9]. Given this innate need for social connectedness, many researchers have considered the relationship between social interactions on social media and individuals’ subjective well-being, as will be discussed below.

### Social media use and subjective well-being

A large body of research has been developed identifying associations between maladaptive use of social media and life contexts such as decreased academic achievement [10] and family functioning [11], and increased appearance concerns [12, 13]. Given the large and broad body of research exploring associations between social media use and a variety of outcomes, the focus of the present literature review and study is the association between social media use and subjective well-being. The construct of subjective well-being encompasses individuals’ evaluations and appraisals of their own lives, including happiness, life satisfaction, and positive affect [14]. Thus, measures of subjective well-being include self-reported satisfaction with life and positive or negative emotionality, or affective well-being [15]. Several studies have investigated associations between social media use and subjective well-being as measured by both satisfaction with life and affective well-being. Regarding satisfaction with life, research has focused primarily on Facebook use, yielding complex and often conflicting results. Among cross-sectional research, a study of Facebook use revealed that individuals who spend more time on Facebook report greater satisfaction with life [16]. In contrast, Błachnio and colleagues found that people who use Facebook with more intensity report poorer satisfaction with life [17]. Other work has shown that, among frequent users of Facebook, feelings of envy incited by the platform were associated with poorer life satisfaction [18]. Among longitudinal research, an experience sampling study of Facebook use in young adults revealed that more Facebook use across a two-week period predicted a subsequent decline in affective well-being [19]. Additionally, an eight-wave study of adolescents revealed small, negative between- and within-person associations of social media use and life satisfaction [20]. Other researchers have taken an experimental/causal approach, for example, examining subjective well-being after participants abstain from social media. In one study, participants who were randomly assigned to abstain from all social media for one week reported declines in life satisfaction and increased loneliness in comparison to participants who continued to use social media [21]. Specific to Instagram use, and in contrast, abstaining from Instagram for one week induced increased life satisfaction and positive affect in a sample of women [22]. Given the above conflicting and context-specific findings, researchers have argued for studies to move away from considering mere time spent on social media and instead focus on patterns of social media use behavior [23], thus highlighting the importance of exploring how social interactions occurring on social media may be influencing subjective well-being.

### Patterns of social media engagement and subjective well-being

When investigating social media use, researchers have attempted to operationalize different types or patterns of use. In one of the first studies to do this, Burke and colleagues [24] classified Facebook use experiences into four categories: directed communication in (e.g., receiving comments and likes), directed communication out (e.g., messages sent, likes given), broadcasting (e.g., posting status updates to one’s online network), and passive consumption (e.g., viewing others users’ profiles). Since then, much research has built upon this, categorizing social media use into active use (collapsing the aspects of directed communication out and broadcasting into a single measure) and passive use, while dropping consideration of directed communication in [25, 26]. Thus, active social media use consists of behaviors that produce information online, such as posting updates, sharing links, or uploading photos, as well as engaging in one-on-one communication such as sending direct messages. In contrast, passive social media use consists of experiences in which information is consumed through browsing news feeds, visiting other users’ profiles, or reading comments [27]. Researchers have capitalized on this active/passive social media use dichotomy, especially when investigating constructs such as subjective well-being.

Initial studies using this active/passive social media use dichotomy appear to demonstrate that more active use is usually associated with greater subjective well-being, while more passive use is usually associated with poorer well-being. For example, a daily diary study of adolescents revealed that more frequent active Facebook use led to increased life satisfaction, while passive Facebook use led to decreased life satisfaction [28]. In addition, a longitudinal study utilizing ecological momentary assessments demonstrated that more passive Facebook use was associated with reduced affective well-being, whereas active use did not impact well-being [25]. A 2017 review by Verduyn and colleagues interpreted this body of research, suggesting that feelings of connectedness resulting from active use are beneficial to subjective well-being, while social comparisons facilitated through passive use are harmful to subjective well-being [27]. Indeed, this interpretation is in line with need to belong theory which supports that humans have evolved to experience social interactions as crucial to health and well-being [5].

Since the publishing of the above review, however, researchers have conducted additional experience sampling and longitudinal research to better understand the implications of active and passive use on subjective well-being, and the findings have been quite mixed. For example, in line with the above study by Verduyn and colleagues, a two-wave study of college students revealed that more passive social media use predicted poorer affective well-being a year later, while no significant associations were found for active use [29]. In contrast, a study conducted among a large, global sample demonstrated that adolescents who engage in more active social media use tend to be less satisfied with their lives [30]. Furthermore, and specific to Instagram use, an ecological momentary assessment study among adolescents demonstrated nonsignificant associations between both active and passive Instagram use and momentary affective well-being [31]. A review of literature published since 2018 summarized that among cross-sectional, longitudinal, and experimental research, significant associations between active use, passive use, and multiple aspects of well-being are rare and often inconsistent with predicted associations [26]. It would appear that, although social connections are evolutionarily vital to individual adjustment and well-being [5], there is much noise in the measurement of social media use and subsequent associations with subjective well-being. Thus, Valkenburg and colleagues concluded in 2021 that researchers should move away from the dichotomy of active and passive self-reported social media use and strive for more complex measurement of observed social media experiences [26].

Beyond a lack of consistent findings related to subjective well-being, an additional critique of the active/passive dichotomy is that there is a lack of consensus in defining active and passive social media use. For example, Valkenburg and colleagues noted a mixture of operationalizations and response scales of active and passive use across studies, with many studies neglecting to include concrete operationalizations all together [26]. This lack of a cross-platform, validated measure of active/passive use has also been noted within additional literature [32], with only a single validated measure for active/passive use developed solely for use of the Facebook platform [33]. More importantly, the active/passive dichotomy does not account for all possible experiences on social media. Indeed, there are additional social media behaviors and experiences that do not fit neatly within the current active/passive dichotomy, such as receiving communications (e.g., likes and messages from others) and monitoring of personal content (e.g., examining one’s own profile), which will be explained in more detail in the next section.

### Multidimensional social media use

The current consideration of active versus passive social media use does not encapsulate the myriad of multidimensional social media uses. For example, as mentioned above, these constructs do not capture inbound communications, which may be related to an individual’s subjective well-being. Interactions on social media are often bidirectional with users both sending and receiving communications (i.e., messages, comments, likes). Specific to Instagram, users primarily receive social communications in the form of likes and comments from others, as the photo-sharing nature of the platform encourages such exchanges. Additionally, users are typically notified of these interactions through push notifications that will alert them to received communications, even while the Instagram app is not in use. Thus, Instagram provides users with constant connection to the inbound communications received on the platform, with such social interactions having implications for subjective well-being. For example, likes are perceived as social support by social media users [34], and receiving a higher number of likes on a social media post has been linked to higher self-esteem [35, 36]. Specific to Instagram users, when people receive more positive social rewards (i.e., number of likes and positive comments) on the app, they report greater subjective well-being [37]. Given humans’ innate need for social interactions [5], such inbound communications are an important aspect of online social interaction which may account for differential associations with subjective well-being. Because researchers do not commonly measure the number of communications received from others when assessing active or passive social media use, they omit this crucial social media experience.

The active/passive social media use dichotomy also fails to capture monitoring of one’s own content, which includes experiences such as looking at personal posts. Passive social media use typically captures non-interactive exploration of *other users’* content via scrolling through newsfeeds and viewing posts shared by others [27]. Therefore, measures of passive social media use have not assessed the monitoring of one’s own personal content while engaging with a social media platform [38]. Instagram, as an image-based platform, may especially encourage monitoring of one’s own content as users can easily scroll through their curated personal profile comprised of carefully selected and shared visual moments. Engagement with one’s own content on social media may be quite relevant to subjective well-being, as viewing one’s own Facebook profile has been linked to higher self-esteem [39]. Similarly, experimental research has found that viewing one’s own personal Instagram profile is associated with higher self-esteem and positive self-perceptions compared to viewing a friend’s or an influencer’s profile [40]. Finally, recalling positive self-presentations posted on Facebook has been found to have a positive effect on subjective well-being [41]. Because the current conceptualization of passive social media use does not include monitoring of one’s own content, associations with subjective well-being remain too broad, as there are multiple forms of passive social media use that could be differentially accounting for this association.

### The present study

In sum, studies examining associations between active and passive social media use and individual’s subjective well-being have demonstrated mixed findings [26, 29–31]. Additionally, literature considering the active/passive dichotomy often contains poor conceptualizations and inconsistent measurement [32], as well as a disproportionate focus on Facebook use [33]. Moreover, the active/passive dichotomy, as currently investigated, fails to account for two specific aspects of social media use: receiving communications and monitoring of personal content, both of which have been related to individuals’ well-being [37, 41]. To address these issues, the aim of the present study was to assess subjective well-being in emerging adults and associate it with objective observational data of their Instagram use. Importantly, the focus was to assess overall *trait* subjective well-being, and thus satisfaction with life was assessed with a validated trait measure, prior to Instagram use. This is in contrast to previous work which has often examined *state* effects of social media experiences, or affective well-being, by measuring acute differences in subjective well-being immediately following social media use [e.g., 19, 25]. This methodology follows suggestions to focus on patterns of engagement with social media rather than time spent on apps [23] and also considers Instagram experiences beyond the active/passive dichotomy (i.e., receiving communications, self-monitoring). In addition, the use of objective observational data reduces bias often associated self-reported social media use [42], and the combination of observational and survey data follows recommendations to use more comprehensive data collection methods [26]. Finally, given that guiding theory states that social interactions are vital to humans’ health and well-being [5], the present study sought to explore which specific types of interactions occurring on Instagram are associated with satisfaction with life.

Unique to this study, participants’ Instagram use was recorded in real time immediately following their posting of a “selfie” portrait. Selfie images are characterized as photos taken of oneself and uploaded to a social media platform [43]. Posting of selfies on Instagram specifically has increased across time [44], with 85% of all shared images on the platform consisting of selfies [37]. Selfie posts have also been found to generate 1.1 to 3.2 times more follower reactions (likes and comments) than non-selfie posts [44]. This context was specifically chosen to ensure opportunities for a wide variety of Instagram experiences and behaviors, especially those beyond the typical active/passive use dichotomy.

Participants’ Instagram behaviors and experiences after posting a selfie image were coded into four mutually exclusive categories: communications received (e.g., number of likes received on posted selfie, number of comments received on posted selfie); communications sent (e.g., number of others’ posts liked, number of comments posted); monitoring self-related content (e.g., number of times checking activity page, number of times viewing personal profile); and exploring other-related content (e.g., number of posts viewed on newsfeed, number of stories viewed). Overall, with the novel and exploratory nature of this study, and due to a lack of consistent findings within previous literature, a single research question was posed: How is overall satisfaction with life associated with the four defined patterns of Instagram experiences following the posting of a selfie?

## Materials and methods

### Participants

Participants were undergraduate students at a large Midwestern U.S. university. Participants were included in the study if they owned an iPhone and used Instagram on their iPhone. Specifically, iPhone users were recruited to ensure compatibility with connection cables and screen recording software (QuickTime) for Mac laptops used in data collection. The final sample size was 70, after excluding 11 individuals because they did not follow directions during the experiment (e.g., did not spend the full 10 minutes using the Instagram app). Participants ranged in age from 18 to 25 years (*M* = 19.8, *SD* = 1.6), with 29 (35.8%) participants identifying as male and 52 (64.2%) identifying as female. The majority of participants identified as White (57.1%) followed by Asian (32.9%), Black or African American (7.1%), and other (2.9%); 4.3% of participants identified as Hispanic or Latino. On average, participants reported spending 2.2 hours a day using Instagram (*M* = 132.0 minutes, *SD* = 202.66).

### Procedure

Ethics approval was obtained from the Michigan State University Institutional Review Board. Participants were recruited using an undergraduate participant pool and received partial course credit as compensation. The present study was not preregistered. Study recruitment began July 9, 2019, and ended March 11, 2020. Interested participants scheduled a time to report to the research lab and complete the study. Participants were asked to come to their appointment with a not-previously-posted selfie image that they were willing to post to their personal Instagram account. Upon arrival to the research lab, participants were seated at a desk with a laptop where they used Qualtrics software to view a digital informed consent document. After reading the informed consent document on the laptop, participants were presented with options to click indicating that they agreed to participate in the study or did not agree to participate; this form of obtaining digital informed consent was approved by the ethics committee prior to data collection. Participants who clicked that they agreed to participate were then directed to the online survey which assessed demographic information and satisfaction with life. Following survey completion, participants connected their iPhone to the laptop so that their iPhone screen could be recorded with QuickTime software. Participants then posted their prepared selfie to their personal Instagram account and were asked to spend 10 minutes using the Instagram app while their phone’s screen was recorded. Participants were instructed not to leave the Instagram app during the 10-minute period and to use Instagram as they normally would during that time. A 10-minute period was chosen to ensure enough time for participants to engage in their typical Instagram use behaviors without inducing fatigue or boredom, as participants were asked to remain only in the Instagram app during the observation time period. Participants were not explicitly asked to refrain from posting another picture during the 10-minute period; however, no participants engaged in further posting activity during the observation period.

Instagram use was recorded immediately after posting a selfie image for several reasons. First, selfie posting presents a context in which users may engage in a wide variety of Instagram experiences such as receiving “likes” and other communications, as well as self-monitoring. These experiences are more likely to occur immediately following a selfie post. Had participants been asked to use Instagram generally, these valuable interactions would likely have been missed, which previous research indicates contribute to subjective well-being [e.g., 37]. Second, it was expected that the lab setting would be restrictive to participants (i.e., not conducive to posting, etc.) and a context was selected in which participants could garner a wider variety of Instagram experiences than would be expected to occur in a lab setting. Third, asking all participants to use Instagram following the uploading of a selfie ensured a consistent context of Instagram interactions among all participants (although see limitations concerning individual differences in Instagram use within the Discussion section). Finally, given that previous research has found positive associations between active social media use and subjective well-being [27, 28], the aim of the present research was to capture associations between satisfaction with life and Instagram experiences within the context of active posting, without which there would be no content to engage with on the platform at all. Important to note, it is impossible to capture all possible types of Instagram behaviors or experiences within a limited lab setting and a short timeframe required for prevention of participant fatigue. Therefore, for the above stated reasons, the present experimental design is optimal, allowing for exploration of the widest possible variety of Instagram experiences.

### Measures

#### Satisfaction with life

Participants completed the 5-item Satisfaction with Life Scale, abbreviated SWLS [45]. Example items: *“In most ways*, *my life is close to my ideal”* and *“I am satisfied with my life”*. Participants responded to each item using a 7-point Likert scale (1 = *strongly disagree*, 7 = *strongly agree*). The mean of each participant’s responses was calculated, with higher scores indicating greater satisfaction with life (*M* = 4.75, *SD* = 1.15). The SWLS has been validated within many populations and across many languages as a measure of the life satisfaction component of subjective well-being [45]. In the present study, the scale demonstrated good internal consistency (α = .81).

#### Demographic characteristics

Participants were asked to report their age, gender, and racial/ethnic identity.

#### Instagram experiences

The screen recordings of participants’ Instagram use were coded by three independent researchers to assess Instagram experiences. A codebook was first developed by having these researchers initially view the same three randomly selected videos and observe participants’ Instagram experiences. This open coding process allowed research team members to note a wide variety of experiences such as checking the activity, newsfeed, and explore pages; viewing user profiles, including their own personal profiles; liking and commenting on posts; receiving likes and comments on posts; and sending and receiving direct messages. Following this open coding, research team members met to discuss observed Instagram experiences and sort the observed experiences into the previously defined categories of: communications received, communications sent, monitoring of self-related content, and exploring other-related content. This resulted in a final codebook to be used for all video recordings (see Table 1).

**Table 1:** List of coded experiences.

**Communications Received**
Number of likes received on posted selfie
Number of comments received on posted selfie
Number of direct messages received
**Communications Sent**
Number of other users’ posts liked
Number of comments posted to other user’s posts
Number of replies to others’ comments
Number of comments liked on any post
Number of direct messages sent
Number of other users’ stories reacted to
**Monitoring Self-related Content**
Number of times opening Activity page
Number of times refreshing Activity page
Number of times viewing own personal profile
Number of times viewing posted selfie
Number of times zooming in on posted selfie
**Exploring Other-related Content**
Number of other users’ stories viewed
Number of other users’ posts viewed on Newsfeed page
Number of ads viewed on Newsfeed page
Number of times refreshed Newsfeed page
Number of times received “You’re all caught up” notification on Newsfeed Page
Number of other users’ posts viewed on Explore page
Number of times scrolling on Explore page without opening a post
Number of novel user profiles opened
Number of posts viewed on other users’ profiles
Number of times viewing comments on other users’ posts

Once the final codebook was created, each video recording was coded by at least two of the three independent coders. Coders entered the number of times each possible experience occurred during the 10-minute recorded Instagram session, resulting in sum count scores for each individual experience. Interrater reliability was determined using intraclass correlations between coders, which were good to excellent for all codes, with mean Pearson’s correlations (*r*’s) within each type of experience ranging from .87 to .98. Composite scores were created for each type of experience, with count scores for each individual experience being summed within each of the four different experience types: communications received (*M* = 11.96, *SD* = 17.76), communications sent (*M* = 9.71, *SD* = 8.80), monitoring of self-related content (*M* = 11.21, *SD* = 10.10), and exploring other-related content (*M* = 124.23, *SD* = 50.74).

### Statistical analysis

Analyses were planned to address our research question. Data cleaning and statistical analyses were conducted using SPSS version 28. Analyses included assessing descriptive statistics, Pearson correlations examining associations between continuous variables, point biserial correlations examining associations between a continuous and a dichotomous variable (i.e., associations with gender), and hierarchical linear multiple regression. To control for possible effects of gender and age, a hierarchical multiple linear regression model was tested. Demographic variables of age and gender were entered as input variables in Step 1 of the model. Step 2 of the model included age, gender, and the four identified Instagram experiences as input variables. In both steps, satisfaction with life was entered as the criterion variable. Standardized regression residuals were normally distributed, and there were no issues with collinearity, thus no assumptions of regression were violated.

### Results

The present study aimed to address the research question: How is overall satisfaction with life associated with various types of Instagram experiences following the posting of a selfie? Correlations among variables are reported in Table 2. Satisfaction with life was positively associated with communications received and exploring other-related content. Additionally, monitoring of self-related content was positively associated with communications received.

**Table 2:** Correlations among variables of interest (*N* = 70).

Variables	1	2	3	4	5	6	7
1. Gender	--						
2. Age	-.19	--					
3. SWLS	.08	-.05	--				
4. Communications Received	.21	-.02	.39**	--			
5. Communications Sent	.24*	-.01	.08	.15	--		
6. Monitor Self-related Content	.14	-.18	.15	.50**	.05	--	
7. Explore Other-related Content	.12	-.06	.30*	.13	.05	.13	--

^a^* *p* < .05

** *p* < .01; SWLS = Satisfaction with Life Scale; associations with gender are included as point biserial correlations, gender coded as 0 = men, 1 = women.

To test for associations between satisfaction with life and Instagram experiences, while accounting for covariates of age and gender, a hierarchical linear multiple regression model was tested (Table 3). The omnibus regression model was significant, *F*(6, 63) = 3.06, *p* = .01, with the chosen correlates explaining 23% of the variance in satisfaction with life scores. The change in *R*^*2*^ between Step 1 and Step 2 was significant, Δ *R*^*2*^ = .22, *F*(4, 63) = 4.43, *p* < .01, indicating that Instagram use experiences explained a significant proportion of the variance in satisfaction with life above and beyond that explained by age and gender. In Step 2 of the model, communications received (ß = .42, *p* < .01) and exploring other-related content (ß = .26, *p* = .03) were positively associated with satisfaction with life. There were no significant relationships between satisfaction with life and either communications sent or monitoring self-related content (*p*’s > .05).

**Table 3:** Unstandardized regression coefficients for effects of predictor variables on satisfaction with life.

Variables	*B*	*SE*	*p*	95% CI
Step 1: *R*^*2*^ = .01, *F*(2, 67) = .28, *p* = .76				
Gender	.18	.29	.54	-.41, .77
Age	-.03	.09	.77	-.21, .15
Step 2: *R*^*2*^ = .23, *F*(4, 63) = 3.07, *p* = .01				
Gender	-.09	.25	.76	-.65, .48
Age	-.04	.08	.64	-.21, .13
Communications Received	**.03**	**.01**	**.003**	**.01, .04**
Communications Sent	.002	.02	.88	-.03, .03
Monitoring Self-related Content	-.01	.02	.44	-.04, .02
Exploring Other-related Content	**.01**	**.003**	**.03**	**.001, .01**

^a^ Change in *R*^*2*^ between Step 1 and Step 2 was significant, Δ *R*^*2*^ = .22, *F*(4, 63) = 4.43, *p* < .01; significant coefficients in bolded text; gender coded as 0 = men, 1 = women.

## Discussion

The present study aimed to objectively and comprehensively measure Instagram experiences following the posting of a selfie. Associations were assessed between the four observed clusters of Instagram experiences and participants’ previously self-reported satisfaction with life. Findings revealed that greater satisfaction with life was associated with receiving more likes and comments (communications received) and higher engagement in passive viewing of other users’ content (exploring other-related content). Of note, data analyses revealed small, yet significant effects, and study findings should be contextualized by this. Satisfaction with life was not significantly associated with sending likes and comments (communications sent) and viewing of one’s own personal content (monitoring self-related content). Interestingly, none of the clusters of Instagram experiences were associated with less satisfaction with life. These findings highlight the importance of both objective data collection and moving beyond the active/passive social media use dichotomy to also consider additional experiences that are self-focused, such as receiving social interactions and monitoring personal content.

Results revealed that participants who were higher in life satisfaction received more communications while using Instagram. This finding aligns with research on social rewards and Instagram use, which has shown that receiving more positive social interactions on Instagram is associated with greater mental well-being [37]. More broadly, receiving social interactions on social media has been associated with higher self-esteem [35, 36]. Importantly, the present methodology moves beyond self-report [37] and perception measures [35, 36] of receiving communications, given the use of objective data and observed real-time receiving of likes and comments. This finding can also be contextualized by need to belong theory which argues that humans have an innate need for social belonging and connectedness, and without these social interactions experience detriments to health and well-being [5]. Thus, the field should move beyond the binary assumption of active versus passive social media use, as findings indicate that receiving communications could be an important factor for understanding social media use and subjective well-being.

Results also revealed that participants who were higher in life satisfaction engaged in more exploration of other’s content on Instagram. This finding appears to contradict some prior research which found that more frequent passive social media use is associated with poorer affective well-being [25] and satisfaction with life [29]. This finding also contradicts an ecological momentary assessment study which did not demonstrate an association between passive Instagram use and affective well-being [31]. There are several potential explanations for the conflicting findings, however. First, objective Instagram use was measured, rather than relying on self-reported measures of social media use, as the previously mentioned studies did. Next, the present study was intentional in considering exploration of other’s content as separate from engaging with or monitoring personal content, a distinction that is not made within the active/passive social media use dichotomy used by these other studies. In other words, these studies captured the passive consumption of both self- and other-created content in their passive measure, while the present multidimensional conceptualization considers the passive consumption of other-related social media content as distinct from monitoring one’s own content. Furthermore, in the present study, subjective well-being was measured with a 5-item scale of trait life satisfaction prior to social media use, while previous research has measured affective well-being with a single state-based item such as, “How do you feel right now? [25]. A final possible explanation for this contradiction may be related to the actual content participants viewed while exploring others’ posts. The present study did not consider the content of posts viewed by participants, instead measuring only the number of posts viewed. Prior literature suggests type of content viewed passively plays a role in determining impacts on subjective well-being. For example, passive viewing of positive posts has been associated with greater positive affect and less negative affect [46]. On the other hand, viewing beauty and fitness posts has been associated with decreased psychological well-being in women [47]. In addition, passive use that is social in nature, meaning users are engaging in passive use to check up on friends or view peers’ posts, has been found to positively influence online well-being [48]. Given that need to belong theory states that social interactions are vital to human well-being [5], perhaps it is these more social types of passive viewing driving the positive association found between exploring others’ content and satisfaction with life in the present study.

Analyses did not reveal a significant association between satisfaction with life and communications sent. Social media behaviors consisting of sending communications are often lumped into the larger conceptualization of active social media use, and nonsignificant associations between active social media use and measures of subjective well-being are not uncommon within the literature. For example, 80% of the published work since 2018 has reported nonsignificant associations between active social media use and various indicators of well-being or ill-being [26]. This null finding does appear to contrast with one research study in which active social media use (i.e., posting, commenting, and liking) was found to be associated with greater satisfaction with life in adolescents [28]. Importantly, similar to the above-stated methodological differences, this prior study did not use objective data and instead asked participants to self-report their daily social media use and considered only active Facebook use. As previously mentioned, the present methodology is unique in that the social media use was measured objectively using real-time observations of actual Instagram use and considered only subjective well-being, rather than affective, state well-being. Most importantly, the present methodology differs from research examining active social media use as the present communications sent measure does not include the posting of content, which is commonly considered to be an aspect of active social media use. Of note, however, although participants in the present study were not explicitly asked to refrain from posting again during the observational period, no participants posted additional images or stories during the 10 minutes.

Finally, there was not a significant association between monitoring self-related content and satisfaction with life. This lack of association is in contrast to previous experimental research which demonstrated that participants’ browsing of their own personal Instagram profile leads to increases in psychological well-being [40]. However, in this prior study, participants completed affectual state measures after spending time viewing their personal Instagram profile. The present study specifically captured *trait* satisfaction with life, measured prior to Instagram use, and associations with various types of Instagram use, including monitoring of one’s own personal content. Additionally, the present measure of monitoring personal content also included behaviors such as checking for follower engagement on their personal activity page, as well as scrutinizing their uploaded selfie image. Importantly, in the present study, self-monitoring behaviors were observed within a novel context: immediately following posting of never-before-uploaded content. Participants were observed immediately after posting a selfie, and only for a 10-minute period, thus creating a unique environment in which participants could monitor appearance of and engagement with freshly uploaded personal content, rather than previously uploaded content. Finally, affective motivations for engaging in self-monitoring may incite differential associations with well-being. For example, self-monitoring to scrutinize one’s own content or engage in upward social comparisons is a vastly different context than monitoring because one is satisfied with or proud of their content, thus associations with subjective well-being may vary given the context of self-monitoring. Future research should continue to investigate the nuance of self-monitoring social media use to further understand associations with subjective well-being.

### Limitations and conclusions

The present study has limitations worth mentioning. First, participants’ life satisfaction was assessed prior to Instagram use. The present study sought to assess trait satisfaction with life, rather than affective, state well-being, and the present methods reflected this. Though the aim was not to attempt to address acute, temporal causality, the order of measurement influences understanding and should be taken into consideration. Future research should address causality in the associations between multidimensional social media use and state/affective well-being. Second, although participants were asked to post a selfie image at the beginning of the observation period, participants did not engage in additional posting during the time period. Posting is a significant aspect of active social media use, and an understanding of active use may be limited by the lack of additional posting by participants. Despite this, participants were still able to participate in active use such as sending and receiving direct messages and liking and commenting on others’ posts. Third, the present study did not account for individuals’ typical selfie posting behavior, with this behavior likely varying among participants (i.e., frequently posting selfies versus rarely posting selfies). As such, the manipulation may have introduced noise in regard to the typical amount of feedback received from participants’ Instagram network. Fourth, all participants were informed during recruitment that posting a selfie was part of the study, therefore, individuals who were uncomfortable with posting a selfie may have self-selected out of the present sample. Thus, additional research is needed to explore observed Instagram use within a variety of contexts, such as posting a non-selfie or not posting at all. Future research may employ experimental designs to explore associations with subjective well-being and Instagram use within a variety of contexts. Fifth, the present study was unable to account for participants’ social media use prior to the study, and we observed Instagram use only within a 10-minute time frame. Participants may have been active on Instagram prior to the study, such as while they waited to participate, which may have influenced engagement during the study period, and observing Instagram activity for only 10 minutes may not have captured the full range of possible behaviors on the app. Future studies may find a way to capture our measures over a more substantial period of time. Finally, the present sample consisted of undergraduate college students using an iPhone to access Instagram. These aspects of the present study should be taken into consideration when contextualizing the present results. Given the limited diversity in participants, findings may not be generalizable to the larger population of Instagram users or individuals who browse Instagram using Android devices. However, provided the high prevalence of social media use among emerging adults [1], understanding social media use in this particular demographic is important. In addition, research could not be found demonstrating that individuals’ use of Instagram varies based on the type of device they use to browse the platform.

In conclusion, the present study employed novel observational methods to improve on previous self-report measurements of social media use and contribute to the growing body of research examining associations between social media use and subjective well-being. Through the use of objective observational methods, the present study was able to capture multidimensional Instagram use experiences, finding that satisfaction with life is positively associated with receiving communications from others and exploring other users’ content. Importantly, the use of objective measures did not confirm previously observed negative relationships between social media use and subjective well-being. Furthermore, the present research supports the idea that future investigation of the topic should continue to move beyond the consideration of mere time spent on social media, as well as the active/passive social media use dichotomy, to consider a broader, more comprehensive range of social media experiences. Future research should consider comparing contexts of social media use (e.g., after posting a selfie versus browsing outside the context of recently uploaded content) and continue exploring healthy social media use and positive associations with subjective well-being. Finally, this understanding of the associations between multidimensional social media use experiences and subjective well-being may help to inform and assist future targeted interventions for problematic social media use, as well as media literacy programming.

## References

[1] Pew Research Center. Social Media Fact Sheet [Internet]. 2021 [cited 2021 Jul 28]. Available from: https://www.pewresearch.org/internet/fact-sheet/social-media/.

[2] Perrin A, Anderson M. Pew Research Center. 2019 [cited 2021 Jul 28]. Share of U.S. adults using social media, including Facebook, is mostly unchanged since 2018. Available from: https://www.pewresearch.org/fact-tank/2019/04/10/share-of-u-s-adults-using-social-media-including-facebook-is-mostly-unchanged-since-2018/.

[3] Auxier B, Anderson M. Pew Research Center. 2021. Social Media Use in 2021. Available from: https://www.pewresearch.org/internet/2021/04/07/social-media-use-in-2021/.

[4] Marengo D, Longobardi C, Fabris MA, Settanni M. Highly-visual social media and internalizing symptoms in adolescence: The mediating role of body image concerns. Comput Human Behav. 2018;82:63–9.

[5] Baumeister RF, Leary MR. The Need to Belong: Desire for Interpersonal Attachments as a Fundamental Human Motivation. Psychol Bull. 1995;117(3):497–529. 7777651

[6] Toyoshima A, Sato S. Examination of the Effect of Preference for Solitude on Subjective Well-Being and Developmental Change. J Adult Dev. 2019;26(2):139–48.

[7] Mair C, Thivierge-Rikard R. The Strength of Strong Ties for older rural adults: Regional distinctions in the relationship between social interaction and subjective well-being. Int J Aging Hum Dev. 2010;70(2):119–43. doi: 10.2190/AG.70.2.b 20405586

[8] Meshi D, Tamir DI, Heekeren HR. The Emerging Neuroscience of Social Media. Trends Cogn Sci. 2015;19(12):771–82. 26578288 10.1016/j.tics.2015.09.004

[9] Ye Y, Lin L. Examining relations between locus of control, loneliness, subjective well-being, and preference for online social interaction. Psychol Rep. 2015;116(1):164–75. doi: 10.2466/07.09.PR0.116k14w3 25621672

[10] Buzzai C, Filippello P, Costa S, Amato V, Sorrenti L. Problematic internet use and academic achievement: a focus on interpersonal behaviours and academic engagement. Social Psychology of Education. 2021;24(1):95–118.

[11] Yan W, Li Y, Sui N. The relationship between recent stressful life events, personality traits, perceived family functioning and internet addiction among college students. Stress and Health. 2014;30(1):3–11. doi: 10.1002/smi.2490 23616371

[12] Caparello C, Verrastro V, Catellani L, Filippello P, Sorrenti L. Maladaptive Instagram Use Among Young Adults: The Mediating Role of Basic Psychological Needs on Body Dissatisfaction. Mediterranean Journal of Clinical Psychology. 2023;11(1).

[13] Wang Y, Xie X, Fardouly J, Vartanian LR, Lei L. The longitudinal and reciprocal relationships between selfie-related behaviors and self-objectification and appearance concerns among adolescents. New Media Soc. 2021;23(1):56–77.

[14] Diener E. Subjective well-being. Psychol Bull. 1984;95(3):542–75. 6399758

[15] Diener E, Oishi S, Tay L. Advances in subjective well-being research. Nat Hum Behav. 2018;2(4):253–60. doi: 10.1038/s41562-018-0307-6 30936533

[16] Gerson J, Plagnol AC, Corr PJ. Subjective well-being and social media use: Do personality traits moderate the impact of social comparison on Facebook? Comput Human Behav. 2016;63:813–22.

[17] Błachnio A, Przepiorka A, Pantic I. Association between Facebook addiction, self-esteem and life satisfaction: A cross-sectional study. Comput Human Behav. 2016 Feb 1;55:701–5.

[18] Krasnova H, Wenninger H, Widjaja T, Buxmann P. Envy on Facebook: A Hidden Threat to Users ‘ Life Satisfaction? In: 11th International Conference on Wirtschaftsinformatik. Leipzig, Germany; 2013.

[19] Kross E, Verduyn P, Demiralp E, Park J, Lee DS, Lin N, et al. Facebook Use Predicts Declines in Subjective Well-Being in Young Adults. PLoS One. 2013;8(8):69841. doi: 10.1371/journal.pone.0069841 23967061 PMC3743827

[20] Orben A, Dienlin T, Przybylski AK. Social media’s enduring effect on adolescent life satisfaction. Proc Natl Acad Sci U S A. 2019;116(21):10226–8. doi: 10.1073/pnas.1902058116 31061122 PMC6534991

[21] Vally Z D’Souza CG. Abstinence from social media use, subjective well-being, stress, and loneliness. In: Perspectives in Psychiatric Care. Blackwell Publishing Inc.; 2019. p. 752–9. doi: 10.1111/ppc.12431 31402459

[22] Fioravanti G, Prostamo A, Casale S. Taking a Short Break from Instagram: The Effects on Subjective Well-Being. Cyberpsychol Behav Soc Netw. 2020;23(2):107–12. doi: 10.1089/cyber.2019.0400 31851833

[23] Timpano KR, Beard C. Social networking and mental health: looking beyond frequency of use and towards mechanisms of action. Neuropsychopharmacology. 2020;45(6):905–6. doi: 10.1038/s41386-020-0629-8 32034280 PMC7265427

[24] Burke M, Kraut R, Marlow C. Social capital on Facebook: Differentiating uses and users. Conference on Human Factors in Computing Systems—Proceedings. 2011;571–80.

[25] Verduyn P, Lee DS, Park J, Shablack H, Orvell A, Bayer J, et al. Passive facebook usage undermines affective well-being: Experimental and longitudinal evidence. J Exp Psychol Gen. 2015;144(2):480–8. doi: 10.1037/xge0000057 25706656

[26] Valkenburg PM, van Driel II, Beyens I. Social Media and Well-being: Time to Abandon the Active-Passive Dichotomy. 2021;0–3.

[27] Verduyn P, Ybarra O, Résibois M, Jonides J, Kross E. Do Social Network Sites Enhance or Undermine Subjective Well-Being? A Critical Review. Soc Issues Policy Rev. 2017;11(1):274–302.

[28] Wenninger H, Krasnova H, Buxmann P. Activity matters: Investigating the influence of facebook on life satisfaction of teenage users. In: ECIS 2014 Proceedings - 22nd European Conference on Information Systems. 2014.

[29] Wang JL, Gaskin J, Rost DH, Gentile DA. The Reciprocal Relationship Between Passive Social Networking Site (SNS) Usage and Users’ Subjective Well-Being. Soc Sci Comput Rev. 2018;36(5):511–22.

[30] Jarman HK, Marques MD, McLean SA, Slater A, Paxton SJ. Motivations for Social Media Use: Associations with Social Media Engagement and Body Satisfaction and Well-Being among Adolescents. J Youth Adolesc. 2021. doi: 10.1007/s10964-020-01390-z 33475925

[31] Beyens I, Pouwels JL, van Driel II, Keijsers L, Valkenburg PM. The effect of social media on well-being differs from adolescent to adolescent. Sci Rep. 2020;10(1):10763. doi: 10.1038/s41598-020-67727-7 32612108 PMC7329840

[32] Trifiro BM, Gerson J. Social Media Usage Patterns: Research Note Regarding the Lack of Universal Validated Measures for Active and Passive Use. Soc Media Soc. 2019;5(2):1–4.

[33] Gerson J, Plagnol AC, Corr PJ. Passive and Active Facebook Use Measure (PAUM): Validation and relationship to the Reinforcement Sensitivity Theory. Pers Individ Dif. 2017;117:81–90.

[34] Rosenthal-von der Pütten AM, Hastall MR, Köcher S, Meske C, Heinrich T, Labrenz F, et al. “Likes” as social rewards: Their role in online social comparison and decisions to like other People’s selfies. Comput Human Behav. 2019 Mar 1;92:76–86.

[35] Scissors L, Burke M, Wengrovitz S. What’s in a Like Attitudes and behaviors around receiving likes on facebook. In: Proceedings of the ACM Conference on Computer Supported Cooperative Work, CSCW. 2016. p. 1501–10.

[36] Wohn DY, Carr CT, Hayes RA. How Affective Is a “like”?: The Effect of Paralinguistic Digital Affordances on Perceived Social Support. Cyberpsychol Behav Soc Netw. 2016;19(9):562–6. doi: 10.1089/cyber.2016.0162 27635443

[37] Maclean J, Al-Saggaf Y, Hogg R. Instagram photo sharing and its relationships with social rewards and well-being. Hum Behav Emerg Technol. 2020 Jul 1;2(3):242–50.

[38] Escobar-Viera CG, Shensa A, Bowman ND, Sidani JE, Knight J, James AE, et al. Passive and Active Social Media Use and Depressive Symptoms among United States Adults. Cyberpsychol Behav Soc Netw. 2018;21(7):437–43. doi: 10.1089/cyber.2017.0668 29995530

[39] Gonzales AL, Hancock JT. Mirror, mirror on my Facebook wall: Effects of exposure to Facebook on self-esteem. Cyberpsychol Behav Soc Netw. 2011;14(1–2):79–83. doi: 10.1089/cyber.2009.0411 21329447

[40] Burnell K, George MJ, Underwood MK. Browsing Different Instagram Profiles and Associations With Psychological Well-Being. Frontiers in Human Dynamics. 2020;2.

[41] Kim J, Lee JER. The facebook paths to happiness: Effects of the number of Facebook friends and self-presentation on subjective well-being. Cyberpsychol Behav Soc Netw. 2011;14(6):359–64. doi: 10.1089/cyber.2010.0374 21117983

[42] Junco R. Comparing actual and self-reported measures of Facebook use. Comput Human Behav. 2013;29(3):626–31.

[43] Deeb-Swihart J, Polack C, Gilbert E, Essa I. Selfie-presentation in everyday life: A large-scale characterization of selfie contexts on instagram. Proceedings of the 11th International Conference on Web and Social Media, ICWSM 2017. 2017;(ICWSM):42–51.

[44] Souza F, De Las Casas D, Flores V, Youn SB, Cha M, Quercia D, et al. Dawn of the selfie era: The whos, wheres, and hows of selfies on Instagram. In: COSN 2015—Proceedings of the 2015 ACM Conference on Online Social Networks. 2015. p. 221–31.

[45] Pavot W, Diener E. The Satisfaction With Life Scale and the emerging construct of life satisfaction. Journal of Positive Psychology. 2008;3(2):137–52.

[46] Choi S, Kim EM. Between Instagram browsing and subjective well-being: Social comparison or emotional contagion? Media Psychol. 2020.

[47] Sherlock M, Wagstaff DL. Exploring the Relationship Between Frequency of Instagram Use, Exposure to Idealized Images, and Psychological Well-being in Women. Psychol Pop Media Cult. 2018;

[48] Chen S, Shao BJ, Zhi KY. Examining the Effects of Passive WeChat Use in China. Int J Hum Comput Interact. 2019;35(17):1630–44.

